# Cloning, Expression and Characterization of a Thermostable Esterase HydS14 from *Actinomadura* sp. Strain S14 in *Pichia pastoris*

**DOI:** 10.3390/ijms160613579

**Published:** 2015-06-12

**Authors:** Pichapak Sriyapai, Fusako Kawai, Somjai Siripoke, Kosum Chansiri, Thayat Sriyapai

**Affiliations:** 1 Department of Biology, Faculty of Sciences, Srinakharinwirot University, Bangkok 10110, Thailand; E-Mail: peechapack@g.swu.ac.th; 2 Center for Nanomaterials and Devices, Kyoto Institute of Technology, Matsugasaki, Sakyo-ku, Kyoto 606-8585, Japan; E-Mail: fkawai@kit.ac.jp; 3 Innovative Learning Center, Srinakharinwirot University, Bangkok 10110, Thailand; E-Mail: somjaisi@swu.ac.th; 4 Department of Biochemistry, Faculty of Medicine, Srinakharinwirot University, Bangkok 10110, Thailand; E-Mail: kosum@swu.ac.th; 5 Faculty of Environmental Culture and Ecotourism, Srinakharinwirot University, Bangkok 10110, Thailand

**Keywords:** *Actinomadura* sp., esterase, α/β-hydrolase superfamily, lipolytic family, thermophile

## Abstract

A thermostable esterase gene (*hydS14*) was cloned from an *Actinomadura* sp. S14 gene library. The gene is 777 bp in length and encodes a polypeptide of 258 amino acid residues with no signal peptide, no *N*-glycosylation site and a predicted molecular mass of 26,604 Da. The encoded protein contains the pentapeptide motif (GYSLG) and catalytic triad (Ser88-Asp208-His235) of the esterase/lipase superfamily. The HydS14 sequence shows 46%–64% identity to 23 sequences from actinomycetes (23 α/β-hydrolases), has three conserved regions, and contains the novel motif (GY(F)SLG), which distinguishes it from other clusters in the α/β-hydrolase structural superfamily. A plasmid containing the coding region (pPICZαA-*hydS14*) was used to express HydS14 in *Pichia pastoris* under the control of the AOXI promoter. The recombinant HydS14 collected from the supernatant had a molecular mass of ~30 kDa, which agrees with its predicted molecular mass without *N*-glycosylation. HydS14 had an optimum temperature of approximately 70 °C and an optimum pH of 8.0. HydS14 was stable at 50 and 60 °C for 120 min, with residual activities of above 80% and above 90%, respectively, as well as 50% activity at pH 6.0–8.0 and pH 9.0, respectively. The enzyme showed higher activity with *p*-nitrophenyl*-*C2 and C4. The *K*_m_ and *V*_max_ values for *p*-nitrophenyl-C4 were 0.21 ± 0.02 mM and 37.07 ± 1.04 μmol/min/mg, respectively. The enzyme was active toward short-chain *p*-nitrophenyl ester (C2–C6), displaying optimal activity with *p*-nitrophenyl*-*C4 (*K*_cat_/*K*_m_ = 11.74 mM^−1^·S^−1^). In summary, HydS14 is a thermostable esterase from *Actinomadura* sp. S14 that has been cloned and expressed for the first time in *Pichia pastoris*.

## 1. Introduction

Lipolytic enzymes, including lipases, triacylglycerol hydrolases (EC 3.1.1.3), esterases, and carboxylester hydrolases (EC 3.1.1.1) are among the most important groups of biocatalysts for biotechnological applications. Bacterial lipolytic enzymes can be classified into eight families based on conserved sequences, pentapeptide motifs (GXSXG) and biological properties [1]. Handrick *et al.* [2] discovered a novel type of hydrolase (PhaZ7) in *Paucimonas* *lemoignei* (formerly *Pseudomonas lemoignei*) that hydrolyzed polyhydroxybutyrate and showed significant homology to lipase LipB from *Bacillus* *subtilis*. Following the lipolytic classification, this new esterase family was designated as family IX. Levisson *et al.* [3] identified a new type of thermostable esterase (EstD) from the hyperthermophilic bacterium *Thermotoga maritima* and classified it into family X. Lee *et al.* [4] screened and identified a novel lipase (LipG) from a metagenomic library of Korean tidal flat sediments, and Kim *et al.* [5] also found a novel cold-adapted alkaline lipase (LipEH166) from a metagenomic library. Additionally, Montoro-Garcia *et al.* [6] identified a new thermostable carboxylase (Est30) from *Geobacillus* *kaustophilus* HTA426, and Rao *et al.* [7] reported a thermostable esterase (EstA3) from *Thermoanaerobacter tengcongensis* MB4, proposing the introduction of novel families (totally 14 families) based on the sequences of EstA3 and other previously reported esterases. These proteins were designated as family XI (LipG), family XII (LipEH166), family XIII (Est30) and family XIV (EstA3).

Esterases and lipases are classified into two groups based on their substrate specificities toward *p-*nitrophenol acyl esters. Esterases hydrolyze the ester bonds of water-soluble acyl esters and emulsified glycerolesters with short-chain acyl groups (≤C8), whereas lipases prefer water-insoluble acyl esters and emulsified substrates with long-chain acyl groups (≥C10) [8]. In addition, cutinases are often categorized as a third group, and these enzymes show a preference for substrates esterified with C4–C8 acyl groups, cutin (a natural polymeric material), and synthetic polyesters [9]. These enzymes all belong to the α/β-hydrolase structural superfamily and exhibit common parallel β-strands surrounded by α-helical connections. These enzymes also contain a catalytic triad formed by serine, histidine, and aspartate/glutamate residues in the polypeptide chain; the active site serine residue is situated at the center of the conserved pentapeptide sequence motif Gly-X-Ser-X-Gly, where X can be any amino acid [10]. Most of lipases possess the complete catalytic machinery, consisting of the catalytic triad and two oxyanion hole residues [11]; the motifs of the first oxyanion hole are classified as either GX or GGGX. Accordingly, a lipase-engineering database was established as a useful tool for protein engineering [11].

Most lipolytic enzymes used in industry do not require cofactors are highly stable in organic solvents, and exhibit a wide substrate specificity and high stereoselectivity [12]. However, enzymes from mesophilic organisms are often unsuitable for industrial applications due to their lack of stability under high temperatures or in detergents and organic solvents. Thus, thermostable enzymes are typically screened from thermophilic or hyperthermophilic organisms [13]. Previously, we isolated a thermophilic actinomycete, *Actinomadura* sp. S14, from rubbish compost in Thailand and cloned a thermostable xylanase gene [14]. To our knowledge, no esterase gene has been cloned from genus *Actinomadura* to date, even though the phenotype of strain S14 on a tributylin agar plate suggested the presence of an esterase.

This study focused on the cloning and characterization of a thermostable esterase from *Actinomadura* sp. S14, HydS14 showed 46%–64% identity and shares a pentapeptide motif with 23 sequences from actinomycetes, but HydS14 was cloned and expressed for the first time among them. The recombinant HydS14 was characterized as a new type of esterase in the lipase superfamily.

## 2. Results and Discussion

### 2.1. Cloning of a Novel Esterase Gene from Actinomadura sp. S14

**Figure 1:**
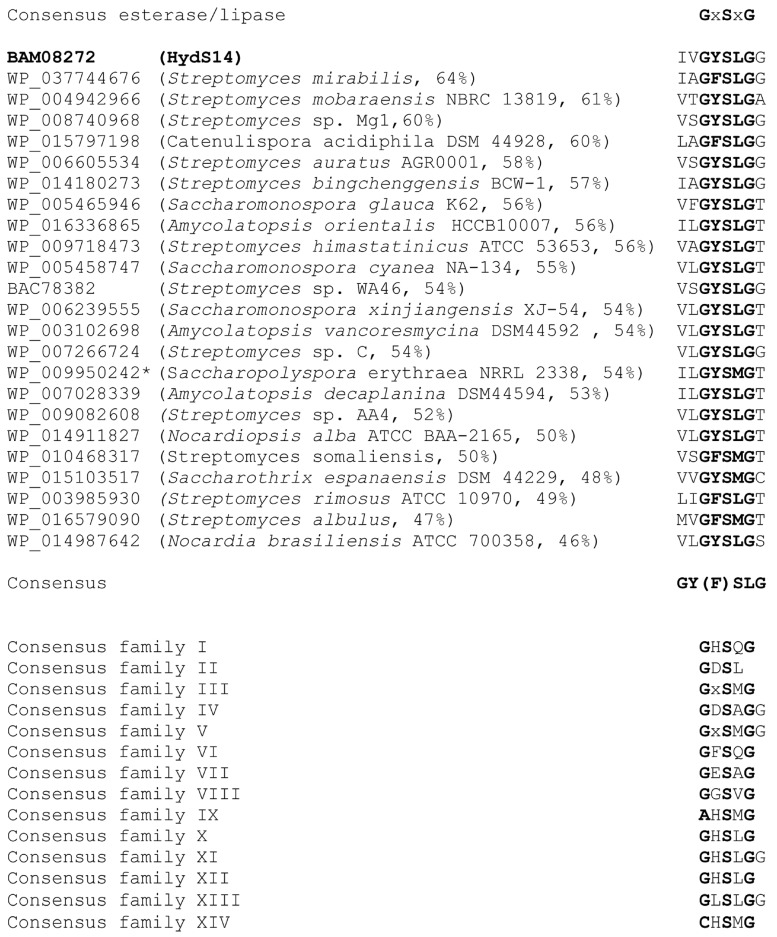
Alignment of the pentapeptide motifs of the HydS14 group with 23 related enzymes and the consensus sequences of 14 known esterase/lipase families Asterisks indicate 3-oxoadipate enol-lactone hydrolases and the other enzymes are α/β-hydrolases. Conserved regions of the pentapeptide motif are shown in bold.

**Figure 2:**
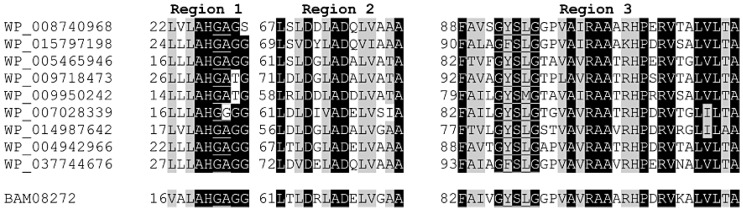
Conserved regions in the HydS14 cluster of putative bacterial lipases. The underlined sequences in region 1 and region 3 correspond to the first oxyanion hole motif and the pentapeptide motif, respectively. The aligned sequences are the representative putative lipases that from the HydS14 cluster: WP_008740968, putative α/β-hydrolase from *Streptomyces* sp. Mg1; WP_015797198, putative α/β-hydrolase from *Catenulispora acidiphila* DSM 44928; WP_005465946, putative α/β-hydrolase from *Saccharomonospora glauca* K62; WP_009718473, putative α/β-hydrolase from *Streptomyces himastatinicus* ATCC 53653; WP_009950242, putative α/β-hydrolase from S*accharopolyspora* *erythraea* NRRL 2338; WP_007028339, putative α/β-hydrolase from *Amycolatopsis decaplanina* DSM44594; WP_014987642, putative α/β-hydrolase from *Nocardia brasiliensis* ATCC 700358; WP_004942966, putative α/β-hydrolase from *Streptomyces mobaraensis* NBRC 13819; and WP_037744676, putative α/β-hydrolase from *Streptomyces mirabilis*. The alignment was performed using the ClustalW program. Identical amino acids are shown with a black background, whereas a gray background indicates identical or equivalent amino acids.

**Figure 3:**
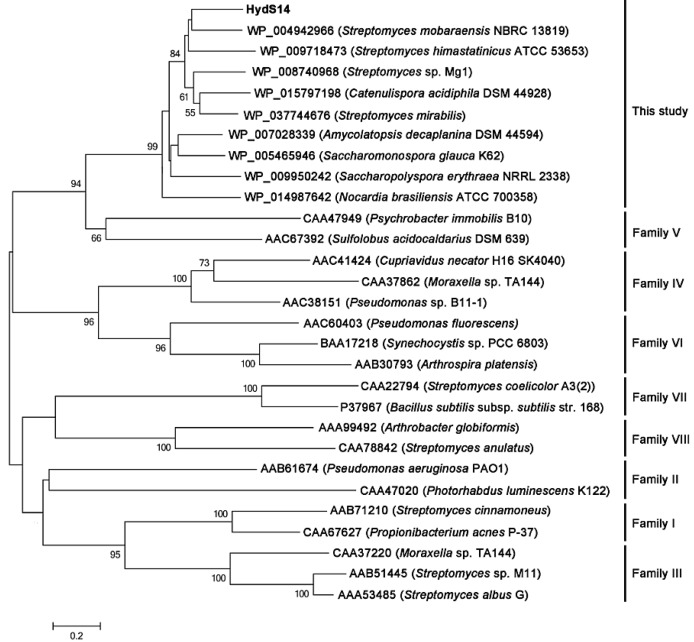
Phylogenetic tree of HydS14 and other esterases/lipase families. The phylogenetic tree was constructed using the neighbor-joining method with the MEGA software, version 4.1. The numbers associated with the branches refer to the bootstrap values resulting from 1000 replicate resamplings; only bootstrap values higher than 50% are shown. The scale represents the number of amino acid substitutions per site. The HydS14 is shown in bold.

The esterase activities of approximately 15,000 transformants from an *Actinomadura* sp. strain S14 genomic library were screened for the formation of a clear zone on low-salt LB agar supplemented with 1% (*w*/*v*) tributylin, 1 mM isopropyl-β-d-thiogalactopyranoside (IPTG) and 50 µg/mL kanamycin, as described in the Section 3.2. Only one transformant showed esterase activity. The positive transformant (pZErO-*S14*) contained an insert of approximately 2.8 kb and the insert’s sequence was deposited into the DDBJ database under accession no. AB562501. The nucleotide sequence of the insert was analyzed using the NCBI sequence analysis tool (http://www.ncbi.nlm.nih.gov/gorf/gorf.html), and it was determined that the pZErO-*S14* insert includes an open reading frame (ORF) of 777 bp (*hydS14*; accession no.: BAM08272 in NCBI (http://www.ncbi.nlm.nih.gov/protein/BAM08272). This ORF encodes a polypeptide of 258 amino acids, with a predicted molecular mass of 26,604 Da. The ORF also possesses a GYSLG motif at positions 86–90, which is in agreement with the consensus sequence motif (GXSXG) of the lipase superfamily. HydS14 showed 46%–64% identity to α/β-hydrolases from a variety of actinomycetes, including genera *Streptomyces*, *Catenulispora*, *Amycolatopsis*, *Saccharomonospora*, *Nocardiopsis*, and *Nocardia*, (Figure 1). All of the sequences contain the conserved GY(F)SLG pentapeptide motif of the lipase superfamily, with the exception of four sequences that have a pentapeptide motif of GY(F)SMG. Taken together, 19 sequences, including HydS14, share the same motif (GY(F)SLG), which is different from the known motifs conserved in the 14 known lipase families. HydS14 possesses the pentapeptide sequence (GYSLG) that is characteristic of the esterase/lipase family. A multiple sequence alignment showed that, with the exception of four proteins with GYSMG (2) and GFSMG (2) motifs, the consensus GY(F)SLG motif is conserved in the α/β-hydrolases from a variety of actinomycetes. A multiple sequence alignment of HydS14 with nine hydrolases from actinomycetes displayed three conserved regions, as shown in Figure 2. These sequences have more than 46% identity with HydS14 (Figure 1) and share three homologous regions in addition to the pentapeptide motif GY(F)SLG (Figure 2). Region 1 includes the predicted first oxyanion hole residue motif GX, where X is an A or G [11]. Taken together, HydS14 can be characterized as a unique group of esterases from actinomycetes, as this motif has not been reported among the 14 families of this group [7]. Therefore, this group has a novel motif and should be considered as the new lipase family. The phylogenetic tree of putative and uncharacterized hydrolases with over 50% bootstrap values to HydS14 is shown in Figure 3. A phylogenic tree of homologous sequences indicated that HydS14 were found to be clustered together with unidentified family of nine representative α/β-hydrolase enzymes from actinomycetes strains and are distinguished from the other lipase/esterases family [1], suggesting that this cluster is a unique group. Recently, new sequences of esterase genes, EstD2 and Est97 have been discovered from metagenomic libraries that were classified into a novel lipolytic enzyme family with close homolog hypothetical enzymes available in the database [15,16]. Although, GxSMG motif is consensus sequence for alpha/beta hydrolase belonging to family III in actinomyces bacteria. However, hydrolase enzymes in family III were not included in this new group cluster. Addition, a BLAST search of HydS14 (BAM08272) revealed 54% identity to 3-oxoadipate enol-lactone hydrolases from *S. erythraea* NRRL 2338 (YP_001103223). Interestingly, 3-oxoadipate enol-lactone hydrolases (EC 3.1.1.24), which are key enzymes in the β-ketoadipate pathway for the dissimilation of aromatic compounds, are included in this same group cluster [17]. α/β-Hydrolase-fold enzymes include various enzyme groups with diverse catalytic functions [18], including diene lactone hydrolases and lipases. HydS14 is the first enzyme of this group that has been cloned and expressed.

### 2.2. Expression and Purification of Recombinant HydS14

**Figure 4:**
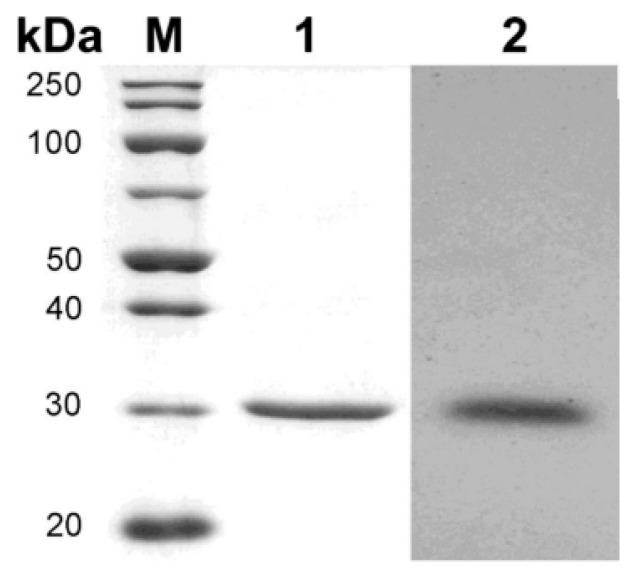
SDS-PAGE and zymography of purified recombinant HydS14. Lane **M**, molecular mass marker; Lane **1**, CBB staining of purified HydS14; Lane **2**, zymogram of purified HydS14.

We cloned the *hydS14* gene that encodes a thermostable esterase from *Actinomadura* sp. S14, an organism from which we previously cloned a gene (*xynS14*) encoding a thermostable xylanase [14]. The expression of a recombinant gene in *E. coli* was negligible. The effective expression of actinomycetes genes in *Pichia* has been previously reported [14]. Because the expression level of a recombinant gene in *Pichia* is dependent on the copy number of the gene in the genome (a higher copy number confers higher resistance to Zeocin), the transformants were screened on yeast extract peptone dextrose (YPD) plates containing 100, 1000, or 2000 µg/mL Zeocin. Twenty positive transformants, from the *P. pastoris* KM71H and X33 strains (which are resistant to 2000 µg/mL Zeocin) were selected and the levels of the expressed protein in the culture supernatants were compared. The esterase activity of the top transformant in strain X33 (218 ± 20 U/mL and 45 ± 1.5 U/mg) was higher than the top transformant in strain KM71H (128 ± 12 U/mL and 18 ± 0.8 U/mg) when grown at 30 °C for 144 h in 2 mL of BMMY medium supplemented with 2% methanol. Therefore, the transformant in strain X33 was used throughout this study. Using SDS-PAGE (Figure 4), the recombinant enzyme was purified to homogeneity (a single band) in two steps and was used for the characterization of the enzyme. The molecular mass was measured to be approximately 30 kDa by sodium dodecyl sulfate polyacrylamide gel electrophoresis (SDS-PAGE), which is in agreement with the predicted value (29,117 Da) for recombinant HydS14. This indicated that *N*-glycosylation did not occur as predicted [19], despite the protein being expressed in *P. pastoris*. SDS-PAGE and zymography showed a single band that was different from recombinant XynS14, which contains a different numbers of glycosyl chains [14]. Together with the absence of *N*-glycosylation site in the ORF, these results suggest that the recombinant HydS14 most likely does not contain glycosyl chains. The purification steps of HydS14 are summarized in Table 1. The enzyme was purified approximately 21-fold, with a yield of 13.8% and a specific activity of 999 ± 25 U/mg.

**Table 1:** Summary of the purification of recombinant HydS14 in *P. pastoris* strain X33.

Purification Step	Total Activity (U)	Total Protein (mg)	Specific Activity (U/mg)	Purification (fold)	Yield (%)
Culture supernatant	2830 ± 110	60	47.2 ± 1.8	1	100
Concentrated supernatant	1270 ± 26	2.2	576 ± 12	12	45
Ni-Sepharose column	390 ± 9.8	0.39	999 ± 25	21	14

### 2.3. Characterization of Recombinant HydS14

**Figure 5:**
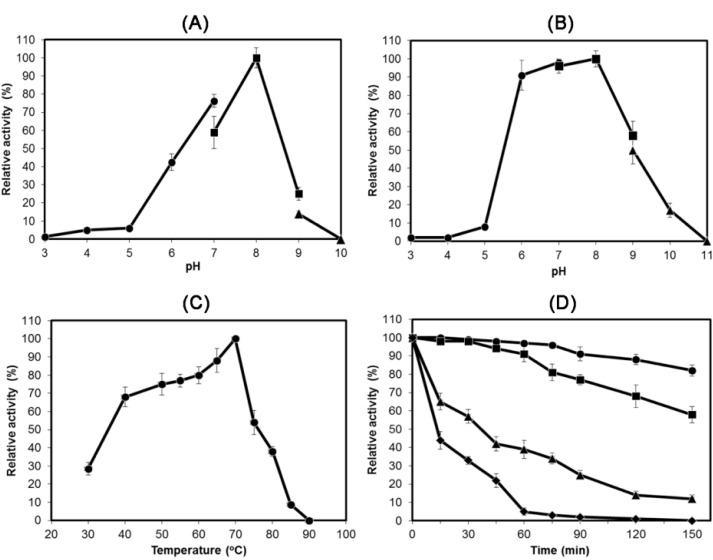
Effects of pH and temperature on the activity and stability of HydS14. (**A**) The optimum pH was determined at 70 °C for 5 min. Filled circles represent citrate phosphate buffer (pH 3.0–7.0), filled squares represent Tris–HCl buffer (pH 7.0–9.0), and filled diamonds represent glycine-NaOH buffer (pH 9.0–10.0); (**B**) The pH stability of the enzyme was determined by measuring the remaining activity after incubating the enzymes at different pH values at 60 °C for 30 min. Filled circles represent 100 mM citrate phosphate buffer, filled squares represent 100 mM Tris–HCl, and filled triangles represent 100 mM glycine-NaOH buffer; (**C**) The optimal temperature was measured in 100 mM Tris–HCl buffer (pH 8.0); (**D**) The thermostability of the enzyme was measured in 100 mM Tris–HCl buffer (pH 8.0). Filled circles represent 50 °C, filled squares represent 60 °C, filled triangles represent 70 °C, and filled diamonds represent 80 °C.

The optimum pH for HydS14 was approximately 8.0, and the enzyme was stable between a pH of 6.0 and 8.0 (Figure 5). No activity was detected at pH values < 5.0 or > 9.0. The optimum temperature for HydS14 was approximately 70 °C, although the enzyme was stable for 120 min at 50 and 60 °C with a residual activity of >80%. However, the stability of HydS14 decreased rapidly at temperatures above 70 °C.

The substrate specificity of HydS14 was determined using *p*NP-acyl esters of straight-chain fatty acids ranging from C2 (acetate) to C16 (palmitate) in chain length as described in the Experimental Section 3.5. The highest activity of HydS14 was shown toward *p*NP-butyrate (C4) (Table 2), and the activity decreased gradually from *p*NP-caproate (C6) to *p*NP-palmitate (C16). No activity was observed for substrates with a chain length of ≥C16. HydS14 exhibited a *K*_m_ of 0.21 ± 0.02 mM and a *V*_max_ of 37.07 ± 1.03 μmol/min/mg for *p*NP-butyrate. The *K*_cat_/*K*_m_ ratio was 11.74 ± 0.78 mM^−1^·S^−1^ and indicated that *p*NP-butyrate (C4) is the best substrate among the *p*NP-acyl ester examined (Table 3). Pleiss *et al.* [11] summarized that the complete catalytic machinery, which contains the catalytic triad and two oxyanion hole residues could be annotated in 91% of the sequence entries in the lipase engineering database, and two sequence signatures, including the first oxyanion hole residues, were identified: GX and GGGX. The GGGX signature includes short chain length-specific lipases and carboxylesterases that have the pentapeptide motif GXSAG. HydS14 contains the GX and GYSLG motifs, even though it is a short chain length-specific lipase and carboxylesterase that acts on C2–C8 *p*NP-acyl esters with a preference for C2 and C4 over C6 and longer. Therefore, HydS14 is clearly an esterase and is not a cutinase (no degradation of polyesters) or a true lipase (no activity toward longer acyl esters).

**Table 2:** Substrate specificity of HydS14.

Substrates	Relative Activity (%)
*p*NP-acyl esters	
*p*NP-acetate (C2)	91 ± 3.5
*p*NP-butyrate (C4)	100
*p*NP-hexanoate (C6)	26 ± 1.2
*p*NP-caprylate (C8)	17 ± 0.8
Triglycerides	
Triacetin	64 ± 2.8
Tributyrin	100
Tricaproin	53 ± 2.5
Tricaprylin	39 ± 1.8

**Table 3:** Kinetic parameter of HydS14 with difference chain lengths *p*-nitrophenyl ester.

Substrates	*V*_max_ (µmol/min/mg)	*K*_m_ (mM)	*K*_cat_ (S^−1^)	*K*_cat_/*K*_m_ (mM^−1^·S^−1^)
*p*NP-acetate (C2)	35.31 ± 1.19	0.36 ± 0.01	2.37 ± 0.06	6.59 ± 0.04
*p*NP-butyrate (C4)	37.07 ± 1.04	0.21 ± 0.02	2.47 ± 0.07	11.74 ± 0.78
*p*NP-caprylate (C6)	42.91 ± 0.77	1.84 ± 0.13	2.86 ± 0.05	1.56 ± 0.08

HydS14 also hydrolyzed triacetin (C2) to tricaprylin (C8) and showed the highest activity with tributyrin (C4). These results indicate that HydS14 is an esterase and not a true lipase. Various detergents and solvents were incubated with HydS14 at final concentrations of 5% and 10% (*v*/*v*) to examine their effects, as described in the Section 3.6, and the residual activities of the enzymes were compared to those of untreated HydS14. The enzyme’s activity remained at above 80% in the presence of 5% Tween 80 or Triton X-100. Greater than 80% of the residual activity remained in the presence of the tested solvents, except for 10% 1-butanol and DMSO (Table 4). The tolerance of recombinant HydS14 for both organic so and nonionic solvents may be useful in industrial applications. The esterase activity was slightly stimulated by the addition of Mg^2+^, K^+^ and Ca^2+^ but was strongly inhibited by Fe^2+^ and Hg^2+^, as shown in Table 5. However, the addition of chelating agents did not significantly alter the enzyme’s activity, suggesting that metal ions are not required for enzyme activity. Although HydS14 does not contain cysteine residues, the enzyme’s activity was inhibited by the addition of a sulfhydryl inhibitor, *p*-chloromercuribenzoic acid. Phenylmethylsulfonyl fluoride (PMSF) strongly inhibited the enzyme’s activity, supporting the hypothesis that the serine residue in the conserved pentapeptide motif is the catalytic amino acid.

The high temperature tolerance and pH stability of HydS14 as well its resistance to mild detergents and polar solvents make it a very useful biocatalyst [20]. Therefore, HydS14 can potentially be used in industrial applications such as oil degumming, removal of pitch from pulp in the paper industry and removal of subcutaneous fat in the leather industry [21,22].

**Table 4:** Effect of detergents and solvents on the HydS14 activity.

Detergent or Solvent	Concentration (% (*v*/*v*))	Relative Activity (%)
None	–	100
Tween 80	5	85 ± 3.5
	10	73 ± 3.5
Triton X-100	5	100
	10	68 ± 3.2
Methanol	5	92 ± 4.5
	10	87 ± 3.9
Ethanol	5	86 ± 4.2
	10	80 ± 3.8
2-Propanol	5	83 ± 4.0
	10	75 ± 3.6
1-Butanol	5	77 ± 3.6
	10	28 ± 1.3
Acetone	5	96 ± 4.6
	10	77 ± 3.6
DMSO	5	91 ± 4.4
	10	55 ± 2.6

**Table 5:** Effects of metals and inhibitors on the HydS14 activity.

Metal or Inhibitor	Relative Activity (%)
None	100
Li^+^	73 ± 3.4
K^+^	115 ± 5.1
Rb^+^	82 ± 4.0
Co^2+^	77 ± 3.6
Mg^2+^	101 ± 4.9
Ca^2+^	105 ± 5.0
Cu^2+^	57 ± 2.4
Ni^2+^	80 ± 3.9
Zn^2+^	64 ± 3.1
Fe^2+^	36 ± 1.7
Mn^2+^	63 ± 3.0
Hg^2+^	27 ± 1.2
*o*-Phenanthroline	91 ± 3.9
*p*-Chloromercuribenzoic acid	87 ± 4.2
EDTA	73 ± 3.6
PMSF	28 ± 1.3
2-Mercaptoethanol	92 ± 4.4

## 3. Experimental Section

### 3.1. Bacterial Strains, Plasmids, Cultivation, and Chemicals

*Actinomadura* sp. S14 [14] was used throughout this paper. *Escherichia coli* TOP 10 cells (Invitrogen, Carlsbad, CA, USA) and pZErO-2 (Invitrogen) were used as the host and vector, respectively, for the construction of a genomic library of strain S14. The pGem^®^-T Easy vector (Promega, Madison, WI, USA), *E. coli* TOP10 F’ cells (Invitrogen), and *E. coli* DH5α cells (Takara Bio, Kyoto, Japan) were used for subcloning. Two expression systems were used for esterase gene cloning and expression: (1) pQE-80L (Qiagen, Valencia, CA, USA) and *E. coli* Rosetta-gami B (DE3) (Novagen, Madison, WI, USA) and (2) pPICZαA and *P. pastoris* strain X33 or strain KM71H using an EasySelect™ Pichia Expression Kit (Invitrogen). *E. coli* Rosetta-gami B (DE3) cells were grown in Luria-Bertani (LB) medium supplemented with 30 μg/mL chloramphenicol at 37 °C for 18–24 h. *P. pastoris* strain X33 or strain KM71H was grown on YPDS agar containing 1% (*w*/*v*) yeast extract, 2% (*w*/*v*) peptone, 2% (*w*/*v*) dextrose, 1 M sorbitol, 100 μg/mL Zeocin and 2% (*w*/*v*) agar according to the provider’s manual. *p-*Nitrophenyl (pNP)-acyl derivatives and glycerol triacyl derivatives were purchased from Sigma-Aldrich (St. Louis, MO, USA). All other chemicals were of the highest grade available through commercial routes.

### 3.2. Construction and Screening of a Genomic Library for Esterase Activity

The chromosomal DNA of strain S14 was extracted following the method of Kieser *et al.* [23], with a slight modification, and was partially digested with *Sau*3AI. Digested DNA fragments of 2–10 kb were extracted using the Gel Extraction Wizard Kit (Qiagen), and these fragments were ligated into *Bam*HI-digested pZErO-2 and transformed into *E. coli* TOP10 cells. The esterase activity of the bacterial transformants was confirmed on low-salt LB agar supplemented with 1% tributylin, 1 mM isopropyl-β-d-thiogalactopyranoside (IPTG), and 50 µg/mL kanamycin. The plates were incubated at 37 °C for 24–48 h, and positive transformants were detected by the formation of a clear zone. The positive colonies were selected, and their activities were reconfirmed using the same procedure. The plasmid harboring the esterase gene was extracted, and the size of the insert was confirmed. The nucleotide sequence of the insert was analyzed by primer walking, and the open reading frame (ORF) was analyzed using the NCBI sequence analysis tool (http://www.ncbi.nlmnih.gov/gorf.html). A homology search against the GenBank database was performed using the BLAST program (http://blast.ncbi.nlm.nih.gov/Blast.cgi), and multiple alignments were performed with the ClustalW program (http://www.ebi.ac.uk/Tools/clustalw2). The ORF of the esterase gene (*hydS14*) suggested that the mature esterase sequence is 777 bp. No signal peptide was predicted using the SignalP 4.1 Server (http://www.cbs.dtu.dk/services/SignalP/). Phylogenetic trees of homologous hydrolase and esterase genes were constructed using the neighbor-joining algorithm in the MEGA 5.1 software with 1000 bootstrap sampling.

### 3.3. Cloning, Expression and Purification

The *hydS14* fragment was amplified by PCR with KOD-Plus DNA Polymerase (Toyobo, Osaka, Japan) in the presence of 5% (*v*/*v*) dimethyl sulfoxide (DMSO) directly from the genomic DNA of *Actinomadura* sp. S14. *Bam*HI and *Pst*I restriction sites were incorporated into the forward and the reverse primer sequences, respectively (forward, 5ʹ-GGATCCAGCAGGTTCGCGCGCACGGT-3ʹ, and reverse, 5ʹ-CTGCAGCTATGCGGCGGTCTCGTCGA-3ʹ; the *Bam*HI and *Pst*I restriction sites are underlined). The following PCR conditions were used: 1 cycle of 5 min at 98 °C, 35 cycles of 15 s at 98 °C, 1 min at 65 °C, and 1 min at 68 °C, and a final extension at 72 °C for 7 min. A poly A tail was added to the PCR products using 10X A-Attachment Mix (Toyobo). The PCR products were then purified and ligated into the pGem^®^-T Easy vector (pGem-*hydS14*). The *hydS14* gene was subcloned into pQE-80L, which was then transformed into *E. coli* Rosetta-gami B (DE3) cells. The transformants were grown in LB supplemented with 50 μg/mL ampicillin at 37 °C for 2–3 h with shaking until the optical density at 600 nm reached 0.5–0.6. IPTG (100 μg/mL) was added to the culture, which was incubated for an additional 12–15 h. The cells were collected by centrifugation and disrupted by sonication. The cell debris was removed by centrifugation, and the resulting cell-free extract was subjected to SDS-PAGE to confirm the expression of HydS14, though no detectable band was observed. We then attempted to express the gene in *P. pastoris.* The *hydS14* gene was amplified using the same procedures as described above except using the primers Hy-XhoF (5ʹ-CCGCTCGAGAAAAGAAGCAGGTTCGCGCGCACGGT-3ʹ) and Hy-XbaR (5ʹ-TGCTCTAGATGCGGCGGTCTCGTCGAGGAACGTTG-3ʹ); the *Xho*I and *Xba*I restriction sites are underlined. The PCR products were ligated into pPICZαA (pPIC-*hydS14*) and transformed into *P.* *pastoris* by electroporation using a Gene Pulser II (Bio-Rad, Hercules, CA, USA) under the following conditions; 1.5 kV, 25 μF, 200 Ω and 4–6 ms. For efficient integration into the *Pichia* genome at the alcohol oxidase (AOX1) locus, pPIC-*hydS14* was linearized with *Sac*I prior to transformation. The digested pPIC-*hydS14* was then purified and transformed into *P. pastoris* KM71H and X33 strains. Transformants from both hosts were grown on YPDS agar containing 100, 500, or 2000 mg/L Zeocin. By this scheme, the promoter for the *AOX1* gene regulates the transcription of the *hydS14* gene, and its expression is induced with methanol. The transformants were cultivated on BMGY medium (1% (*w*/*v*) yeast extract, 2% (*w*/*v*) peptone, 1.34% (*w*/*v*) yeast nitrogen base, 0.1 M potassium phosphate (pH 6.0), 0.4 μg/mL biotin, and 1% glycerol) at 30 °C for 48 h with shaking until the optical density at 600 nm reached 2–6. The yeast cells were aseptically harvested by centrifugation at 1200 rpm for 10 min, resuspended in 2 mL of BMMY medium (1% (*w*/*v*) yeast extract, 2% (*w*/*v*) peptone, 1.34% (*w*/*v*) yeast nitrogen base, 0.1 M potassium phosphate (pH 6.0), 0.4 μg/mL biotin, and 0.5% methanol) and incubated at 30 °C for 6 days with shaking. At 24 h intervals methanol was added to the culture to maintain a final concentration of 0.5%. The culture of each of transformant was centrifuged at 5000× *g* for 20 min to remove the cells. The supernatants were concentrated using a Vivaspin 20 concentrator (Sartorius, Tokyo, Japan) with a 10 kDa molecular weight cut-off membrane filter, and the resulting concentrate was absorbed onto a Ni-Sepharose 6 Fast Flow column (GE Healthcare, Tokyo, Japan). The enzyme was eluted with 50 mM Tris–HCl buffer (pH 8.0) containing 300 mM imidazole HCl. The enzyme was desalted using PD-10 columns (GE Healthcare) and subjected to SDS-PAGE to confirm the purity of the recombinant protein.

### 3.4. SDS-PAGE and Zymography

SDS-PAGE was performed using 12.5% polyacrylamide geld according to the method of Laemmli [24]. The samples were dissolved in 6× sample buffer and heated in a boiling water bath for 5 min. After electrophoresis, the proteins were visualized by Coomassie Brilliant Blue R-250 (CBB) staining. For zymography, SDS was removed from the SDS-PAGE gel by gentle shaking in 25% (*v*/*v*) isopropanol at 4 °C for 1 h, and the gel was then washed twice with distilled water to remove the isopropanol [25]. The washed gel was overlaid with 1% (*w*/*v*) tributyrin agar gel containing 100 mM Tris–HCl buffer (pH 8.0) and kept at 60 °C for 3 h. Activity in a resolved band was observed as a clear zone against the white background of the gel but is shown as the reverse image in Figure 4.

### 3.5. Determination of Esterase Activity

Esterase activity was determined by measuring the absorbance of *p-*nitrophenol liberated from the *p*-nitrophenyl-butyrate (C4) substrate at 410 nm. The enzyme reaction was performed at 50 °C for 5 min in 50 mM Tris–HCl buffer (pH 8.0) containing 0.4% Triton X-100, and 0.1% gum arabic. A final concentration of 0.2% SDS was added to terminate the reaction and put on ice. One unit of esterase activity was defined as the amount of enzyme that produced 1 μmol of *p*-nitrophenol per min at 50 °C. The extinction coefficients of *p*-nitrophenol were determined for every condition prior to each measurement. The background hydrolysis of the substrate was measured in a reference sample without the enzyme. The amount of protein was determined using the Bradford method [26]. The substrate specificity of the enzyme was determined using various *p*-nitrophenyl-acyl esters with different acyl chains (*p*-nitrophenyl-acetate (C2) to *p*-nitrophenyl-caprylate (C8)). All the experiments were performed in triplicate. The kinetic parameters were calculated using Lineweaver-Burk plots.

The activity of the esterase toward glycerol triacyl derivatives (triacetin, tributyrin, tricaproin and tricaprylin) was determined using a standard titration assay, with minor modifications [27]. The substrate emulsion (25 mL of 5% (*w*/*v*) concentration) was preincubated at 60 °C for 15 min with magnetic stirring in a 50 mL flask with a stopper. After the addition of the purified enzyme, the total volume was adjusted to 30 mL, and 5 mL of each reaction mixture was withdrawn every 5 min. This aliquot was added to 25 mL titration cocktail flasks containing 10 mL of 95% (*v*/*v*) ethanol and 2–3 drops of 1% (*w*/*v*) thymolphthalein. Titration was performed with a burette titrator using 0.05 N NaOH until a light blue color appeared. The liberated fatty acids were calculated based on the amount of NaOH used to reach the titration end point using the following Equation:

μmol fatty acid/mL = [(mL of NaOH for sample − mL of NaOH for blank) × 0.05 × 1000]/5

A reaction curve was created by plotting the quantity of liberated fatty acid over the time of the reaction, and the activity of the enzyme was determined from the slope of the linear portion. One unit was defined as the amount of enzyme that produced 1 μmol of fatty acid per min under the specified assay condition. The reaction was performed in duplicate for each substrate.

### 3.6. Characterization of Recombinant HydS14

The optimum pH was measured using three different buffers at a final concentration of 100 mM with a pH range of 3.0–10.0 (citrate phosphate buffer for pH 3.0–7.0, Tris–HCl buffer for pH 7.0–9.0 and glycine-NaOH buffer for pH 9.0–10.0) at 70 °C for 5 min. The optimum temperature was determined in 100 mM Tris–HCl buffer (pH 8.0) at different temperatures (30–90 °C). The pH stability was determined by measuring the remaining activity after incubating the enzyme at different pH values at 60 °C for 30 min. The purified enzyme was incubated at different temperatures from 50 to 80 °C for 150 min in 100 mM Tris–HCl buffer (pH 8.0), and the residual activity was measured. The effects of detergents and solvents on the esterase activity were determined by incubating the enzyme at 37 °C for 30 min in 100 mM Tris–HCl buffer (pH 8.0) containing 5% or 10% (*v*/*v*) of detergent or solvent. The residual activity was measured by comparing the enzyme activity in the presence of detergent or solvent to the enzyme activity in the absence of detergent or solvent. The effects of inhibitors and metal ions on esterase activity were measured by determining the residual activity after incubating the enzyme at 37 °C for 1 h with 1 mM of each inhibitor or metal ion. The enzymatic activity without any additive was defined as 100%. All the assays were performed in triplicate using *p-*NP-butyrate, as described.

## 4. Conclusions

A novel esterase gene (*hydS14*) was cloned from an *Actinomadura* sp. S14 gene library. The encoded protein (consisting of 258 amino acids) contains a pentapeptide motif (GYSLG) and the catalytic triad (Ser88-Asp208-His235) of the esterase/lipase superfamily. HydS14 showed 46%–64% identity to 23 sequences from actinomycetes, including three conserved regions and a novel (GY(F)SLG) motif that distinguished the cluster from other clusters of the α/β-hydrolase structural superfamily. HydS14 with a 6X His tag (expressed in the plasmid pPICZαA-*hydS14*) was inducibly expressed with methanol in *Pichia pastoris* under the control of the AOXI promoter in a secreted protein form, consisting of a single protein that was not *N*-glycosylated. HydS14 demonstrated optimum activity at a temperature of approximately 70 °C and a pH of 8.0. The enzyme was stable at 50 and 60 °C for 120 min, with residual activity of more than 80% and over 90% and 50% activity in pH ranges of 6.0–8.0 and pH 9.0, respectively. HydS14 was tolerant to detergents and solvents, suggesting a potential for industrial applications.
